# Downregulation of the *INDEHISCENT* Gene by RNAi Resulted in Desired Pod Shatter Reduction of *Lepidium campestre* in Subsequent Generations

**DOI:** 10.3390/ijms242115943

**Published:** 2023-11-03

**Authors:** Emelie Ivarson, Annelie Ahlman, Jan-Eric Englund, Ida Lager, Li-Hua Zhu

**Affiliations:** 1 Department of Plant Breeding, Swedish University of Agricultural Sciences, P.O. Box 190, SE-234 22 Lomma, Swedenida.lager@slu.se (I.L.); li-hua.zhu@slu.se (L.-H.Z.); 2 Department of Biosystems and Technology, Swedish University of Agricultural Sciences, P.O. Box 190, SE-234 22 Lomma, Sweden; jan-eric.englund@slu.se

**Keywords:** *Brassica* species, field cress, oilseed and catch crop, RNAi, pod shatter, *IND* gene

## Abstract

Wild species field cress (*Lepidium campestre*) has favorable agronomic traits, making it a good candidate for future development as an oil and catch crop. However, the species is very prone to pod shatter, resulting in severe yield losses. This is one of the important agronomic traits that needs to be improved in order to make this species economically viable. In this study, we cloned the *L. campestre INDEHISCENT* (*LcIND*) gene and prepared two *LcIND*-RNAi constructs with the *IND* promoter (long 400 bp and short 200 bp) from Arabidopsis. A number of stable transgenic lines were developed and evaluated in terms of pod shatter resistance. The majority of the transgenic lines showed increased resistance to pod shatter compared to the wild type, and this resistance was maintained in four subsequent generations. The downregulation of the *LcIND* gene by RNAi in the transgenic lines was confirmed by qRT-PCR analysis on T_3_ lines. Southern blot analysis showed that most of the analyzed lines had a single-copy integration of the transgene, which is desirable for further use. Our results show that it is possible to generate stable transgenic lines with desirable pod shatter resistance by downregulating the *LcIND* gene using RNAi in field cress, and thus speeding up the domestication process of this wild species.

## 1. Introduction

Dispersal of seeds is the end point of successful fertilization and fruit development. However, premature fruit dehiscence or pod shatter can result in huge yield losses in canola; for instance, losses of between 11 and 25% have been reported [1], but up to 50% of the yield can be lost if harvesting is delayed [2]. Apart from lost harvests, seeds that are prematurely shattered and fall to the ground might germinate, which has major implications for volunteer control in subsequent crop cultivations in the field [3]. Therefore, pod shatter resistance is an important target for the improvement of oilseed crops.

For fruit dehiscence to occur in *Brassica* species *Arabidopsis thaliana*, correct formation of specific tissues within the fruit (also called the silique or pod) is crucial. Morphologically, a silique consists of two fruit valves, which contain two seeds separated by a thin structure called the septum. The outer margin of the septum, called the replum, is connected to the valves by valve margins or dehiscence zones (DZs) with two layers: the lignified cell layer and the separation layer, which consists of small spherically shaped parenchyma cells [4]. At fruit maturity, the parenchyma cells secrete cell-wall-degrading enzymes to degrade the middle lamella, generating a breaking zone [5]. When the fruit ripens, it dries and shrinks, and the structures that are lignified remain rigid, building up tension within the fruit. The pressure forces the valves to separate from the replum at its weakest point, namely the breaking zone of the separation layer, generating fruit (pod) shatter [6].

The establishment of a functional DZ has been shown to be associated with several transcription factors (TFs) [4,7,8]. The MADS-box-domain TFs *SHATTERPROOF1* (*SHP1*) and *SHATTERPROOF2* (*SHP2*) are expressed in the DZ, where they trigger the expression of basic helix–loop–helix (BHLH) genes *INDEHISCENT* (*IND*) and *ALCATRAZ* (*ALC*) [4,9]. Furthermore, they are involved in the development of the valve margin [8]. *IND* is important for the formation of both the lignified cell layer and the separation layer [9], while *ALC* is vital only in the development of the separation layer [4]. Correct fruit patterning depends on the exclusive expression of these genes in the DZ. MADS box gene *FRUITFULL* (*FUL*) is partially involved in differentiation and expansion of valve cells and negatively regulates the expression of the *SHP1*/*SHP2* genes in the valve [7,10]. Homeodomain transcription factor *REPLUMLESS* (*RPL*) restricts the expression of *SHP1*/*SHP2*, *IND* and *ALC* from the replum side [11]. It has been shown that this genetic network interacts with the activities of auxin and gibberellin (GA) in Arabidopsis [12,13,14] Local depletion of auxin and the biosynthesis of GA are required at the valve margin for pod shatter to occur. For instance, *IND* mediates these events by directly inducing the expression of *GA4*, a GA biosynthesis gene [14]. The production of gibberellin leads to depression of *ALC*, a key regulator of valve margin formation.

By downregulating or overexpressing genes involved in the dehiscence network, pod shatter resistance has been achieved in several *Brassica* species. Ostergaard et al. [3] ectopically expressed the *AtFUL* gene in *Brassica juncea*, resulting in transgenic lines with pod-shatter-resistant fruits due to the absence of valve margin specification. RNAi silencing of *BnSHP* in *B. napus* generated plants with drastically reduced *BnSHP* expression and non-shattered pods when ripened [15]. By overexpressing a *MADSB* gene from mustard in winter rapeseed, Chandler et al. [16] obtained plants that did not shatter due to altered carpel development. Liljegren et al. [8] showed that the mutation of both *SHP1* and *SHP2* genes in *Arabidopsis* resulted in pods that did not shatter due to the absence of DZ in the mature fruits. RNAi silencing of the *LcIND* gene in *L. campestre* using the CaMV 35S promoter resulted in transformants with reduced dehiscence capacity [17]. However, total encapsulation of the seeds in a fruit with no remaining ability to dehisce is undesirable for oilseed crop production, as significant losses would be incurred when attempting to retrieve the seeds [18]. Therefore, it is desirable to increase the pod shatter resistance, while it is also important that the pod shatter resistance not be so strong that it prevents pod shatter during post-harvest threshing.

Field cress (*Lepidium campestre*) is a wild species within the Brassicaceae family with positive agronomic traits and a biennial nature, making it a potential candidate as a future oil and catch crop. However, it is prone to pod shattering, a problem that needs to be solved in order to make this species economically viable. The replum-valve region of the fruits of field cress is patterned very similarly to the dehiscent fruits of *A. thaliana* [19]. Previous studies [3,17,20] have revealed that the fruit developmental and molecular pathways underlying pod shatter are highly conserved among Arabidopsis and other *Brassica* species. However, the fruits of field cress are shorter and wider than those of Arabidopsis and are therefore called silicles instead of siliques. In this study, in order to develop new field cress breeding lines with a reasonable degree of pod shatter resistance, namely not shattering before harvest, yet not too hard to open for threshing, we downregulated the *L. campestre INDEHISCENT* (*LcIND*) gene under the *AtIND* promoter using RNAi. The results show that stable transgenic lines with desirable degrees of pod shatter resistance could be obtained by downregulating the *LcIND* gene using RNAi, demonstrating that the use of RNAi is an efficient approach for the regulation of pod shatter resistance in field cress, thus speeding up the domestication process of the species.

## 2. Results and Discussion

Pod shattering is essential in the reproduction and survival of wild species for the next generation. However, in cultivation, premature pod shattering is a serious problem that can cause considerable yield losses. In the history of plant domestication for agricultural production, one of the first traits improved was pod shattering. As a wild oil species, field cress has some positive agronomic characteristics, making it a promising candidate for domestication as a future oil crop. Since it is winter-hardy and biennial, it is also a valuable candidate as a catch crop for cold regions in the future. In order to develop field cress into an economically viable crop, pod shattering needs to be controlled. In this study, we investigated the possibility of reducing pod shattering in this species through genetic modification. The results show that it is possible to regulate pod shattering through downregulation of one key gene controlling the formation of the dehiscence zone.

Molecular mechanisms underlying pod shattering have been studied extensively in recent years, and some key genes associated with the process have been identified. In an effort to improve pod shatter resistance and perform functional analysis of the target genes, transgenic lines with fruit indehiscence have been obtained in several crop species through modification of target gene expression levels [3,4,8,15,16,17]. In this study, the *LcIND* gene in field cress was downregulated by the *AtIND* promoter using RNAi, resulting in fruits less prone to dehiscence compared to wildtype (WT) fruits.

### 2.1. Confirmation of Transgenic Lines

**Figure 1:**
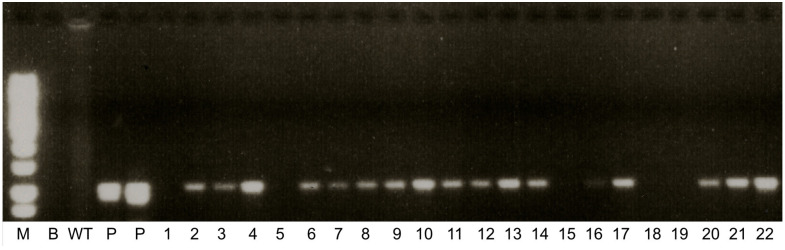
PCR results showing the integration of the *nptII* gene in the transgenic lines of *L. campestre*. Lanes 1–22, putative transgenic lines; WT, wildtype; M, molecular markers (100 bp); B, blank; P, plasmid DNA.

**Figure 2:**
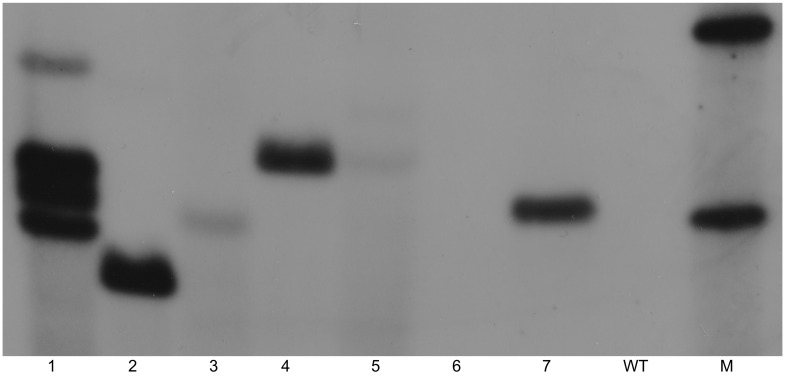
Determination of copy number using Southern blot analysis of some T_1_ transgenic lines (lanes 1–7) and WT of *L. campestre*. M = DIG-labelled molecular markers. The DNA was digested with the *Bgl*II enzyme and hybridized with DIG-labeled *nptII* probe.

The transformation events yielded a number of putative transgenic lines in which the transgene integration was initially confirmed by PCR analysis. Figure 1 shows the partial PCR result of the analyzed T_1_ lines. Further confirmation of the stable integration into the plant genome and the copy number of transgene *nptII* was obtained by Southern blot hybridization in some T_1_ lines. Among the analyzed lines, the copy number of the *nptII* gene ranged from one to four, with most of the lines having single-copy integration, namely line 1 with four copies; line 5 with two copies; and lines 2, 3, 4 and 7 with single-copy integration (Figure 2). It is desirable to develop transgenic lines with single transgene integration for commercial purposes in order to maintain the stability of improved traits and to avoid any potential gene silencing by multiple copies of transgene integration [17].

### 2.2. Gene Expression Level

**Figure 3:**
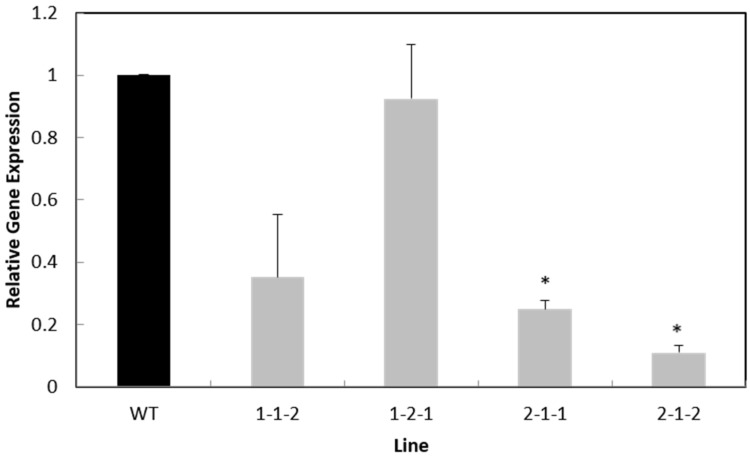
Quantification of *LcIND* gene expression in immature pods of T_3_ transgenic lines 1-1-2, 1-2-1, 2-1-1 and 2-1-2, along with WT, using qRT-PCR analysis. The results are presented as the means of three biological replicates per line and three technical replicates per biological replicate. Error bars represent standard deviation (SD), and lines marked with * are significantly different from the WT at *p* = 0.05.

**Figure 4:**
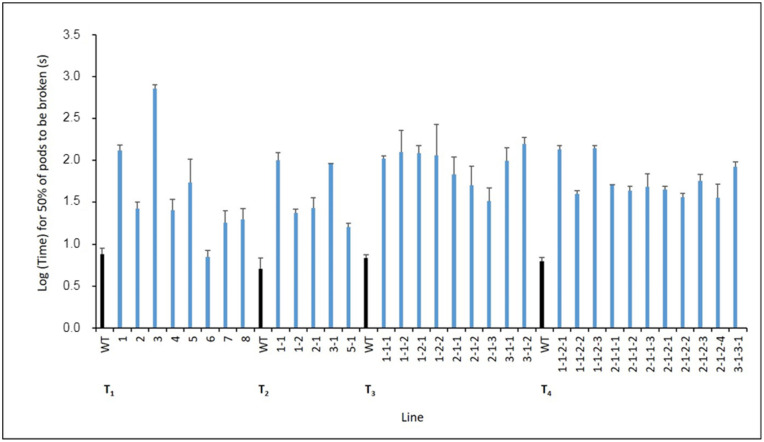
Half-life dehiscence for WT and transgenic lines from four generations (T_1_–T_4_) as determined by the random impact test and expressed as time on a logarithmic scale (log10). Lines 1 and 8 harbored a vector with a 400 bp promoter, while lines 2–7 harbored a vector with a 230 bp promoter. The T_1_ generation was agitated at 9 Hertz, while T_2_–T_4_ were agitated at 15 Hertz. The results are presented as means of three replicates per line (with minor exceptions for missing values) and 3 × 3 replicates for WT. Error bars represent standard deviation (SD) and the pooled standard deviation for WT. All lines are significantly different from WT at *p* = 0.05, except for line 6 in the T_1_ generation.

The expression level of the *LcIND* gene was analyzed through quantitative reverse transcription-PCR (qRT-PCR) in four T_3_ transgenic lines. The results showed that the *LcIND* gene expression was slightly downregulated (by 7.6%) in one of the four lines but highly downregulated in the remaining three lines by 70%, 69.6% and 86.7%, respectively, with a significant difference in two lines compared to the WT (Figure 3). The two lines with a significant reduction in *LcIND* gene expression were transformed with the pWG-*LcIND*-230 vector, indicating that various lengths of the *AtIND* promoter might affect downregulation of the *LcIND* gene differently. Further studies are needed to confirm this. However, our results do not show a clear correlation between the expression level of the *LcIND* gene and the increase in pod shatter resistance (Figure 4). This is not consistent with the results obtained by Lenser and Theissen [17], who reported that the reduction in *LcIND* gene expression correlated well with an increase in dehiscence half-life. However, in that study the *LcIND-*RNAi gene was under the control of the 35S promoter. It is well known that 35S is a constitutive and strong promoter, and it is expected that the gene expression under 35S promoter would be stronger than that under the native *IND* promoter. However, in our case, we did not want a strong downregulation of the *IND* gene in order to avoid a complete block of pod shattering.

### 2.3. Pod Shatter Resistance

**Figure 5:**
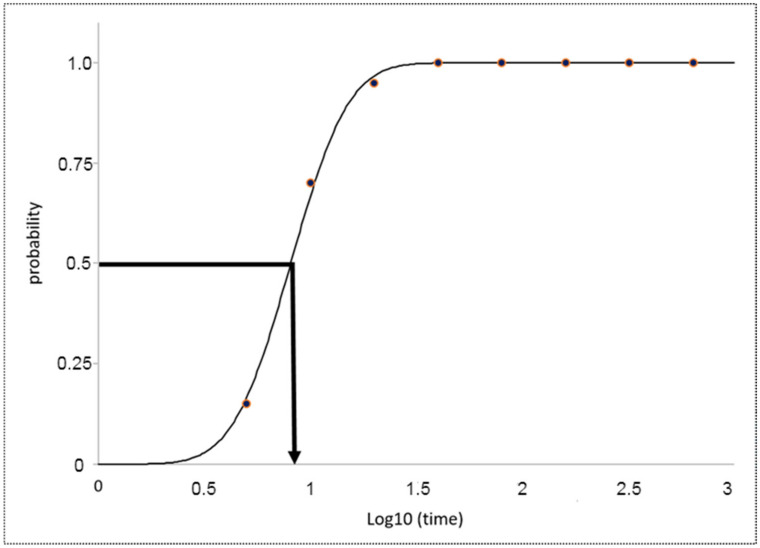
Illustration of the estimated half-life dehiscence (in this case, for one replicate of the WT in the T_4_ generation). The red points are observed values, the black curve line is the estimated line while the strong black horizontal and vertical line with the arrow illustrates the point where 50% of pods were opened.

The effect of *LcIND-*RNAi downregulation on pod shattering was analyzed through a random impact test, similar to the method described by Bruce et al. [21]. The analysis revealed varying levels of pod shatter resistance in the transgenic lines already shown clearly in T_1_, with silicles from the most resistant lines requiring a considerable amount of force to shatter the seeds compared with the WT. Also, the difference in pod shatter between the WT and the transgenic lines was visible even before the analysis, in the biotron, where a substantial portion of the WT pods had already opened up, while the pods of the transgenic plants were still tightly closed. In T_1_, the agitation force was set to 9 Hertz. The half-life of dehiscence, i.e., the time needed to open up half of the pods, expressed as time on a logarithmic scale (Figure 4 and Figure 5), for the WT plants ranged between 5.7 and 12.1 s, while the transgenic lines showed a half-life ranging from 6.2 to 815.2 s. The lines with varying degrees of pod shatter resistance were further evaluated in the subsequent generations. The agitation force was increased to 15 Hertz for T_2_–T_4_ plants in order to reduce the working time required for evaluation.

The half-life dehiscence analysis for WT plants in the T_2_, T_3_ and T_4_ generations showed a half-life ranging between 2.4 and 11.1 s, while the half-life of dehiscence from the transgenic T_2_ lines varied from 14.8 to 126.5 s. Both intermediate and highly pod-shatter-resistant lines from the T_2_ generation were maintained in the T_3_ generation. In T_3_, the half-life of dehiscence spanned from 25.5 to 308.1 s. Again, both highly and intermediately pod-shatter-resistant lines were maintained in the T_4_ generation. In T_4_, the analysis of half-life dehiscence showed values ranging between 23.7 and 149.2 s (Figure 4).

The decreased dehiscence capacity was maintained through all four generations in all tested transgenic lines of field cress, as shown in Figure 4, indicating stable transgene integration and an efficient downregulating effect of the *LcIND*-RNAi gene. We also proved that both lengths of the *AtIND* promoter functioned well in increasing the pod shatter resistance in field cress.

For commercial production, a maximum level of resistance to pod shatter may not be desirable, while a certain degree of resistance might be preferred, where the fruits do not dehisce before harvest but are ready to shatter when needed. In this study, we obtained stable transgenic lines with different degrees of pod shatter resistance, some of which may be suitable for further breeding and cultivation purposes in the future.

Downregulation of the *IND* gene has been shown to affect fruit morphology. It has been reported that the fruits of *Arabidopsis* mutated in the *IND* gene lacked a margin definition. Neither the small cells of the separation zone nor the lignified layers were apparent in the *ind* fruits [9]. Similar results were achieved when the *LcIND-*RNAi gene under the control of the 35S promoter was expressed in *L. campestre* by Lenser and Theissen [17]. Microscopy studies revealed altered fruit patterning in transgenic indehiscent fruits, with both the separation layer and the lignified layer being absent. Also, the replum was heavily flattened in some of the transgenic fruits.

In this study, we did not visually observe morphological changes in fruits of the transgenic plants in general, except for a few plants from line 2 in the T_1_ generation, which were found to be sterile. tThe sterility does not appear to correlate with the downregulation of the *LcIND* gene, as the majority of the transgenic plants from this line flowered and set seeds normally.

In conclusion, we developed transgenic lines of field cress with indehiscent fruits by expressing *LcIND-*RNAi under the partial sequences of the *AtIND* promoter. This improved trait was shown to be stably maintained in the subsequent four generations. The successful application of RNAi for the production of commercially desirable lines with reduced pod shatter resistance in field cress demonstrates the considerable potential of this technique in speeding up the domestication of this wild species as a future oil and catch crop.

## 3. Materials and Methods

### 3.1. Plant Material

Field cress (*Lepidium campestre*) seeds with accession number NO94-7 were used in this study. The genotype was originally collected by late Professor Arnulf Merker in Öland, Sweden, and the seeds were obtained from plants grown in a greenhouse.

### 3.2. Transformation Vectors

In this study, we used two different lengths of the *IND* promoter from *Arabidopsis thaliana* in order to obtain transgenic lines with desirable pod shatter resistance. Genomic DNA from *A. thaliana* Col-0 was used to amplify 400 bp and 230 bp regions of the *IND* promoter with the same forward primer but different reverse primers (Supplementary Table S1). The 230 and 400 bp promoter fragments were then cloned into Gateway vector pWatergate (CSIRO, Canberra, Australia) using the restriction sites *Mss*I and *Avr*II, respectively, thereby replacing the existing promoter in the vector. A 300 bp fragment in the middle of the *IND* gene from *L. campestre* (FJ907544.1) was chosen as a target sequence for the two *LcIND*-RNAi constructs. The fragment was amplified from cDNA of *L. campestre* using the primers listed in Supplementary Table S1 and cloned into the pDONR^TM^221 vector using the Gateway^®^ system (Thermo Fisher Scientific, Gothenburg, Sweden). The RNAi fragment was then transferred to the pWatergate vector containing an *A. thaliana IND* promoter of either 230 bp or 400 bp in length. The vectors contain the *nptII* gene for plant selection. The two RNAi constructs were named pWG-*LcIND*-230 and pWG-*LcIND*-400. The ready-to-use vectors were transformed into the *Agrobacterium* strain AGL-1 for plant transformation.

### 3.3. Transformation

The hypocotyls were used for transformation. The disinfection and germination of the seeds, as well as plant transformation, were carried out according to Ivarson et al. [22]. Briefly, hypocotyl explants were precultured on MS [23] medium supplemented with 30 g L^−1^ sucrose, 2.5 g L^−1^ agar and 0.5 mg L^−1^ 2.4-D at pH 5.7 for 2 days prior to transformation. The explants were soaked in an O/N-cultured bacterial suspension, followed by coculture under light for 4 days on MS medium with 30 g L^−1^ sucrose, 2.5 g L^−1^ agar and 1.1 mg L^−1^ TDZ at pH 5.7. Excess *Agrobacterium* left on the explants was rinsed with MS20 (MS with 20 g L^−1^ sucrose) for 1 min before they were placed on selection medium. The composition of the selection medium was the same as for the coculture medium, except for supplementation with 150 mg L^−1^ ticarcillin and 15 mg L^−1^ kanamycin. The kanamycin concentration was increased to 25 mg L^−1^ in the second subculture and further raised to 30 mg L^−1^ in the subsequent subcultures. The cultures were kept in light for the whole experimental period. Regenerated shoots of about 1 cm in size were transferred to the shoot proliferation medium according to Li et al. [24] and Li et al. [25].

### 3.4. In Vitro Culture, Plant Growth Conditions and Management

The in vitro cultures were grown in a controlled climate chamber with a photoperiod of 16 h at 33 µmol m^−2^ s^−1^ and a temperature of 21/18 °C (day/night).

The plants were vernalized at 4 °C for 8 weeks in vitro in order to induce flowering, then grown in the biotron until harvest. The plants were cultured in a growth chamber under conditions of a 16 h photoperiod at 250 µmol m^−2^ s^−1^ light intensity, 21/18 °C (day/night) and 60% humidity. The plants were regularly watered and fertilized with long-lasting fertilization granules (N:P:K = 21:3:10).

### 3.5. Pod Shatter Resistance Analysis

Pod shatter resistance was analyzed by the random impact test according to the method reported by Lenser and Theissen [17] and Bruce et al. [21], with minor adjustments. Mature and dry pods equivalent to developmental stage 19 for Arabidopsis [26] were randomly collected from both wild-type and transgenic lines of field cress cultivated in the growth chamber with a humidity of 60%. Each plant was analyzed with 3 replicates and 20 pods per replicate. The pods were placed together with six 4 mm metal balls in a 50 mL grinding jar of a MM 400 mixer mill (Retsch GmbH, Haan, Germany) and shaken in intervals of 5, 10, 20, 40, 80, 160 and 320 s or until all pods were open for all generations. After each interval, the jars were opened, and the number of non-opened pods was counted. In T_1_, the agitation force was set to 9 Hertz, while for the T_2_–T_4_ generations, the agitation force was altered to 15 Hertz in order to reduce the duration of the evaluation work.

### 3.6. Half-Life of Dehiscence

To estimate the half-life of dehiscence from the random impact test, a method similar to the one described by Bruce et al. [21] was used. The likelihood function was used, assuming a probit model, to estimate the maximum likelihood of the half-life of dehiscence. This method turned out to be more robust with less bias than the one described by Bruce et al. [21].

### 3.7. PCR Analysis

Putative transgenic shoots grew on the selection medium for a minimum of eight weeks before they were analyzed by PCR. The total genomic DNA from the shoots grown in vitro was extracted by the CTAB method [27]. Successful integration of *nptII* was analyzed by PCR using the primers listed in Supplementary Table S1. The PCR reactions were carried out according to Zhu and Welander [28].

### 3.8. Southern Blot Analysis

Southern blot hybridization was conducted for further confirmation of successful transgene integration, along with determination of the copy number of the transgenes. Around 20 μg of genomic DNA extracted from the putative in vitro shoots by the CTAB method [27] were digested with restriction enzyme *Bgl*II. The non-radioactive DIG system was used in Southern blot hybridization [29]. The same primers as for the PCR analysis were used to synthesize the probe for the *nptII* gene.

### 3.9. Quantitative Reverse Transcription PCR Analysis

Immature seeds and pods from the T_3_ generation plants were collected 28–35 days after flowering, and total RNA was extracted using an RNeasy Plant Mini Kit (Qiagen, Hilden, Germany). With the exception of the addition of 4% polyvinylpyrrolidone (PVP) to the extraction buffer, the protocol provided by the manufacturer was followed. Between 80 and 100 mg of plant tissue was used for each sample, with three biological replicates for each line and three technical replicates for each sample in the qRT-PCR reaction. The samples were treated with DNase in order to remove possible traces of the genomic DNA using TURBO DNA-free kit (Ambion, Austin, TX, USA). In a 20 μL reaction, 1000 ng of total RNA was used to synthesize the first-strand cDNA using Superscript III First-Strand Synthesis Supermix for qRT-PCR (Invitrogen, Life Technologies, Carlsbad, CA, USA). The synthesized cDNA was diluted 5×, and 6 μL was used for each 20 μL qRT-PCR reaction, using a BIO-RAD C1000 Thermal Cycler and a CFX 96 Real-Time System with BIO-RAD iQ SYBR Green Supermix (Bio-Rad, Hercules, CA, USA). The following PCR program was used: 95°C for 10 min followed by 40 cycles of 95 °C for 15 s, 63 °C for 30 s and 72 °C for 30 s. Melting curve analysis was included in the qRT-PCR analysis to confirm product specificity. The primer pair for the *IND* gene was chosen since it had proved to work well as reported by [17] (Supplementary Table S1). The expression of the *IND* gene was normalized towards the *TIP41*-like reference gene, which was previously proven to be a reliable reference gene in field cress by Lenser and Theissen [17], Muhlhausen et al. [30], and Ivarson et al. [31] (Supplementary Table S1).

### 3.10. Statistical Analysis

For qRT-PCR analysis, data were analyzed with ANOVA and Dunnett’s test, with the significance level set at *p =* 0.05 using the Minitab statistical program, version 19.2020.1.

For half-life dehiscence analysis, a one-way ANOVA of the logarithm (log10) of the estimated half-life times was used to compare different lines. The data transformation was performed to better satisfy the assumptions of normality and homoscedasticity. Contrasts were used to compare the mean of the WT lines with the transgenic plants. The significance level for the comparison was *p =* 0.05, and Holm’s adjustment was used for each generation to compensate for the number of comparisons. For the analysis, the emmeans package in R was used.

## Data Availability

All data analyzed during this study are included in this published article.

## Supplementary Materials

The supporting information can be downloaded at: https://www.mdpi.com/article/10.3390/ijms242115943/s1.
