# Supplementary material for: Downregulation of the INDEHISCENT Gene by RNAi Resulted in Desired Pod Shatter Reduction of Lepidium campestre in Subsequent Generations
Source: Int J Mol Sci. 2023 Nov 3;24(21):15943. doi: 10.3390/ijms242115943 (PMC10650181; doi:10.3390/ijms242115943)
Supplement: Supplementary file 1 [file ijms-24-15943-s001.zip › ijms-2674368-supplementary.pdf]

Table S1 Sequences of the primers used in the study.

Gene	Forward Primer *(5′-3′)*	Reverse Primer *(5′-3′)*	Purpose
AtIND promoter (230 bp)	GTTTAAACTGGGCAATACCTTAATGTCG	CCTAGGCTAAACCCCACGGTTTCTTCG	Cloning
AtIND promoter (400 bp)	GTTTAAACTGGGCAATACCTTAATGTCG	CCTAGGGCCATAAGGAAGCATAAGTCG	Cloning
LcIND gene fragment	GGGGACAAGTTTGTACAAAAAAGCAGGCTGATGAAGACATGGATGCCATGA	GGGGACCACTTTGTACAAGAAAGCTGGGTTCCAAGGTGAGAGTGAGGCTGAA	Cloning
nptII	GCCCTGAATGAACTGCAGGACGAGGC	GCAGGCATCGCCATGGGTCACGACGA	PCR
LcTIP41-like	GCTTATGAGATTGAGAGAGACGAGAA	GGATACCCTTTCGCAGATAGAGAC	qRT-PCR
LcIND	TAAGCGACGATCCTCAGACG	AAACATTGCGGTGGGATTGC	qRT-PCR
